# A Systematic Review of the Effects of Smoking on the Cardiovascular System and General Health

**DOI:** 10.7759/cureus.38073

**Published:** 2023-04-24

**Authors:** Mihirkumar P Parmar, Mankirat Kaur, Sravani Bhavanam, Gopi Sairam Reddy Mulaka, Lyluma Ishfaq, Roopeessh Vempati, Mohammed Faseel C, Hima Varsha Kandepi, Rajagopal ER, Sweta Sahu, Shubha Davalgi

**Affiliations:** 1 Internal Medicine, Gujarat Medical Education and Research Society (GMERS) Medical College, Vadnagar, Vadnagar, IND; 2 Internal Medicine, Sri Guru Ram Das Medical College and Hospital, Amritsar, IND; 3 Internal Medicine, Sri Devaraj Urs Medical College, Kolar, IND; 4 Physiology, St. Martinus University Faculty of Medicine, Willemstad, CUW; 5 Medicine, Directorate of Health Services Kashmir, Srinagar, IND; 6 Internal Medicine, Gandhi Medical College and Hospital, Hyderabad, IND; 7 Internal Medicine, Government Medical College Kozhikode, Kozhikode, IND; 8 Medical Education, NRI Medical College of Science, Guntur, IND; 9 Internal Medicine, Bicol Christian College of Medicine, Albay, PHL; 10 Surgery, Jagadguru Jayadeva Murugarajendra (JJM) Medical College, Davangere, IND; 11 Community Medicine, Jagadguru Jayadeva Murugarajendra (JJM) Medical College, Davangere, IND

**Keywords:** effects on general health, cardiovascular system, effects of smoking, cigarette smoking, smoking tobacco

## Abstract

The main risk factor for atherosclerotic cardiovascular disease is smoking. Nicotine and carbon monoxide are two dangerous substances that are found in cigarette smoke. The increased heart rate can have an almost instantaneous impact on the heart and blood vessels. Smoking is well known to cause oxidative stress, endanger the lining of the arteries, and accelerate the accumulation of fatty plaque in the blood vessels. It raises the danger of sudden thrombotic events, inflammatory alterations, and low-density lipoprotein oxidation. The smoke's carbon monoxide decreases the blood's capacity to deliver oxygen, adding to the heart's stress. Notably, these risks increase when diabetes, hypertension, high cholesterol, and glucose intolerance are present. It has a detrimental effect on peripheral blood vessels, raising the possibility of thromboangiitis obliterans. Stroke risk is known to be increased by smoking. As compared to those who continue to smoke, those who give up smoking have a much longer life expectancy. Chronic cigarette smoking has been shown to affect the macrophages' ability to remove cholesterol. Abstinence from smoking enhances the function of high-density lipoproteins and cholesterol efflux, lowering the risk of plaque buildup. In this review, we present the most recent information regarding the causal relationship between smoking and cardiovascular health as well as the long-term advantages of quitting.

## Introduction and background

Smoking is a widespread and compulsive behaviour that has been associated with a number of health issues, including lung illness, cancer, and heart disease. One of the worst side effects of smoking is cardiovascular disease, and the chemicals in tobacco smoke can harm the heart and blood vessels, causing atherosclerosis, coronary heart disease, stroke, and other cardiovascular disorders. Despite the fact that smoking is recognised to be unhealthy, millions of individuals still smoke. As a result, smoking is the top preventable cause of death globally. Many studies have looked at how smoking affects health in general, the heart, and blood vessels. Our review gives a useful summary of the existing literature, evaluates the evidence base critically, points out knowledge gaps, and makes suggestions for future research. Our review could help shape future research, public health policies, and interventions that aim to reduce the number of people who smoke and the health risks that come with it. Given the significant impact of smoking on cardiovascular health and the urgent need for effective smoking cessation interventions, a review of this nature would be a valuable contribution to the field of tobacco control and cardiovascular health.

Inhaling smoke from burning tobacco, whether in the form of cigarettes, cigars, or pipes, is a prevalent but dangerous practice. It is a common behaviour that is the world's biggest cause of mortality and is avoidable. Cigarette smoke (CS) is made up of gases as well as tar-like liquid and solid particles. These compounds are poisonous, mutagenic, and carcinogenic, and can have other detrimental impacts on health. Nicotine, tar, carbon monoxide, ammonia, formaldehyde, acrolein, acetone, polyaromatic aromatic hydrocarbons (PAHs), hydroxyquinone, nitrogen oxides, and cadmium are a few of the particular chemicals found in CS [1]. Smoking can harm the heart, lungs, reproductive system and bones, and potentially cause cancer. It can also harm many other body systems. Smoking can have an effect on the cardiovascular system, which can lead to coronary artery disease, peripheral vascular disease, an aortic aneurysm, hypertension, and stroke [2-4]. Cigarette smoke contains more than 7,000 toxic substances, including nicotine, tar, and carbon monoxide. These substances increase heart rate and contractility, cause inflammation, impair endothelial function, cause thrombus formation, and lower blood levels of high-density lipoprotein cholesterol, all of which are associated with the development of cardiovascular diseases [5]. Asthma and chronic obstructive pulmonary disease (COPD) are two lung diseases that can be caused by smoking. It can cause pulmonary vascular endothelial cells to die, which makes COPD more likely to happen. The pulmonary vascular endothelial cells are important for maintaining the function of blood vessels, and their death can contribute to the pathogenesis of COPD [6].

Also, smoking can affect the reproductive system, which can lead to foetal deaths, stillbirths and trouble in getting pregnant. It can also increase the risk of oral cavity problems like periodontitis [7]. Furthermore, smoking increases the risk of cancer [8,9]. Tobacco smoke is a complex mix of chemicals that includes many mutagens and carcinogens, such as polycyclic aromatic hydrocarbons (PAHs) and tobacco-specific nitrosamines (TSNAs), which are found in all tobacco products, including cigarettes, cigars, and smokeless tobacco. When tobacco is burned or heated it releases these chemicals into the air, where they can be inhaled or ingested by smokers and non-smokers alike. Once inside the body, these chemicals can cause changes to the DNA in cells, leading to mutations and the uncontrolled growth of abnormal cells, which can ultimately result in cancer [10]. Even though everyone knows smoking is bad for health, a lot of people still do it and find it hard to stop because nicotine is so addictive. In this review, we talk about how smoking affects the heart and overall health.

Methods

**Figure 1:**
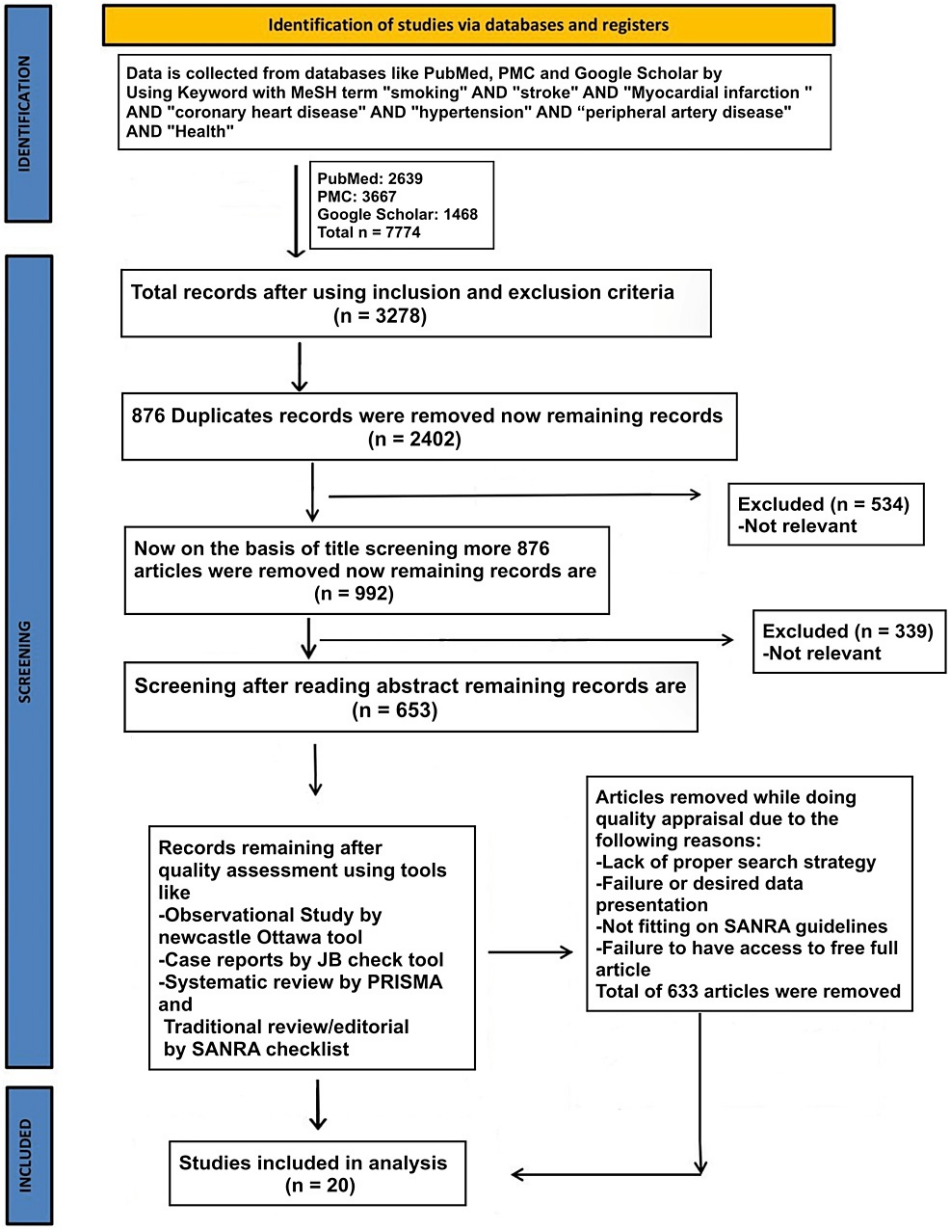
PRISMA flow chart PRISMA: Preferred Reporting Items for Systematic Review and Meta-Analysis; MeSH: Medical Subject Headings; SANRA: scale for quality assessment of narrative review articles; PMC: PubMed Central

We conducted our systematic review in accordance with the Preferred Reporting Items for Systematic Reviews and Meta-Analysis (PRISMA) criteria (Figure 1) [11]

We used the following medical subject headings (MeSH) terms with keywords like "smoking" AND "stroke" AND "Myocardial infarction" AND "coronary heart disease" AND "hypertension" AND "peripheral artery disease" AND "Health" to gather data from the National Library of Medicine (PubMed), PubMed Central (PMC), and Google Scholar. There were 7774 items altogether that were located in electronic databases.

Inclusion and Exclusion Criteria

In our analysis, we looked at all full-text papers, studies with people as subjects, and papers that were published in English. There were papers about smoking's effect on general health and the cardiovascular system published in the last 20 years, from 2002 to 2022 including clinical trials, controlled clinical trials, randomised controlled trials, observational studies, case-control studies, prospective cohort studies, population-based cohort studies, and cross-sectional studies were included.

We didn't include research that didn't involve people or articles that didn't have the full text. Systematic reviews, meta-analyses, and clinical reviews were not considered.

Results

The papers were reviewed by each author, and those that were not relevant to our topic were manually removed. Tables 1-2 provide a summary of studies.

**Table 1:** A list of studies which shows the effects of smoking on the cardiovascular system MI: myocardial infraction; CHD: congenital heart defects; HP: haptoglobin; PAD: peripheral arterial disease; MACE: major adverse cardiovascular events; NRT: nicotine replacement therapy; NCAD: non-compressible arterial disease; CAD: coronary artery disease; CVD: cardiovascular disease; PPCI: primary percutaneous coronary intervention; STEMI: ST-elevation myocardial infarction; DES: drug-eluting stent

Citation	Year of publication	WHO region	Country of the study	Focus of the study	Findings	Key observation	Disease
Matsuo et al. [2]	2020	Western Pacific region	Japan	To clarify the relationship between smoking habits and functional results following an ischemic stroke.	Those who gave up smoking within two years of having a stroke had a greater odds ratio for a poor functional outcome. (1.75 [1.15–2.66] versus nonsmokers). For present smokers, the likelihood of a poor functional outcome appeared to rise with daily cigarette consumption (P for trend=0.002).	At three months following an acute ischemic stroke, smoking is linked to a higher risk of poor functional outcomes.	Stroke
Sun et al. [12]	2021	Western Pacific region	China	To investigate the association between smoking and significant outcomes in patients who had undergone thrombolysis as part of the international quasi-factorial randomised Enhanced Control of Hypertension and Thrombolysis Stroke Study (ENCHANTED).	A smoker's health is less advantageous than a nonsmoker's.	Young smokers had a higher risk of developing CVD than non-smokers did.	Stroke
Reinstadler et al. [13]	2017	European region	Germany	Among patients receiving primary percutaneous coronary interventions (PPCI) for ST-elevation myocardial infarctions (STEMI), to assess the association between smoking and myocardial salvage and damage as well as clinical outcomes.	When compared to non-smokers, smokers had considerably reduced rates of MACE (3.8 vs. 8.5%, P = 0.01) and death (0.9 vs. 3.9%, P = 0.01).	MACE risk in patients with two or more defective coronary arteries is double that of people with just one damaged vessel.	MI
Patti et al. [14]	2016	European region	Italy	After myocardial infarction (MI) and drug-eluting stent (DES) implantation, patients receiving various oral P2Y12 antagonists were investigated for the effects of smoking on platelet reactivity.	Platelet reactivity increased after smoking (from 165±17 at T1 to 170±17 at T2, P = 0.0002).	Smoking and oral antiplatelet medication effectiveness are moderate and unlikely to have clinical impacts.	MI
Alotaibi et al. [15]	2021	European region	United Kingdom	Smoking causes decreased postprandial metabolism and is a known independent risk factor for coronary heart disease.	Triacylglycerol and C-reactive protein levels after meals are higher in smokers than non-smokers (primary effect group effect size [Cohen's d] = 0.94, P = 0.034).	In the early postprandial interval (0–4 h), exercise-induced reduction in postprandial triacylglycerol was higher in nonsmokers than in smokers (21%, d = 0.43, vs 5%, d = 0.16, respectively; group-condition interaction P = 0.061).	CHD
Chen et al. [3]	2019	Western Pacific region	China	The purpose of the study was to examine how patients with coronary artery disease's high-density lipoprotein function after quitting smoking.	Smokers with coronary artery disease showed a considerable improvement in high-density lipoprotein's antioxidative and antichemotactic properties after three months of quitting.	Smoking cessation did not increase the cellular cholesterol efflux caused by high-density lipoproteins.	CHD
Aung et al. [4]	2019	Southeast Asian region	Thailand	The study's goal was to determine how high-density lipoproteins in patients with coronary artery disease behave when they stop smoking.	Participants in the intervention arm quit smoking at a rate that was significantly greater than those in the control arm (25.62% vs 11.32%; adjusted odd ratio 2.95; 95% confidence interval 1.55-5.61).	Participants who received the evidence-based intervention package had a success rate for quitting smoking that was about three times higher than participants who received the standard care.	HP
Schwartz et al. [16]	2015	Region of the Americas	USA	By adding Panel Management Assistants (PMAs) to primary care teams with and without panel management training, it will be possible to evaluate the efficacy of integrating panel management into clinical practice.	In comparison to patients on control teams, patients on intervention teams had higher odds of receiving NRT (OR = 1.4; 95% CI 1.2-1.6) and enrolling in the disease management services MOVE! (OR = 1.2; 95% CI 1.1-1.6) and Telehealth (OR = 1.7, 95% CI 1.4-2.1).	Decrease in hypertension following smoking cessation	HP
Song et al. [17]	2015	Western Pacific region	China	Effects of smoking cessation in individuals with vascular diseases on macrophage cholesterol efflux	Smokers with NCAD and CAD had significantly lower plasma apoA-1 and HDL-cholesterol (HDL-C) levels than nonsmokers (p = 0.002, p 0.001, and p = 0.019 and p = 0.004, respectively).	Smokers with NCAD had higher apoB and triglyceride levels than nonsmokers (p = 0.003 and p = 0.038, respectively).	PAD
Rajaee et al. [18]	2019	Region of the Americas	USA	Smoking cessation intervention for patients with vascular disease: a standardized approach	16 patients (31%) had totally stopped smoking, and 40 patients (77%) had cut back by at least 50%.	After quitting smoking, the severity of vascular disorders is reduced.	PAD

**Table 2:** A list of studies which shows smoking effects on general health SD: standard deviation, HR: hazard ratio, CI: confidence interval, fMRI: functional magnetic resonance imaging, MMSE: mini-mental state examination, CPD: cigarettes per day

Citation	Year of publication	WHO region	Country of the study	Focus of the study	Findings	Key observation	Disease
Perski et al. [19]	2020	European region	United Kingdom	We evaluated the self-reported levels of physical discomfort among current daily smokers, never-daily smokers, and former daily smokers, divided by age group, and controlled for a broader variety of covariates than has previously been done, such as health status, neuroticism, anxiety, and depression.	Reported levels of bodily pain in former daily smokers were higher than in never-smokers in 16- to 34-year-olds, and reported levels of bodily pain were also higher in former daily smokers than in never-daily smokers in those aged 35-64 years.	Body pain is more common in everyday smokers than in nonsmokers of all ages.	Bodily pain
Petre et al. [20]	2015	Region of the Americas	USA	The connection between smoking, the development of chronic pain, and brain physiology was examined.	Patients with acute and chronic back pain were more likely to smoke, although there was no correlation between smoking and pain intensity. Smoking was a significant predictor of subacute back pain 1 year after the onset of symptoms, which is largely attributable to the synchronicity of fMRI activity between two brain regions. (the nucleus accumbens and the medial prefrontal cortex).	Smoking increases the chance of developing chronic back pain, and the corticostriatal circuitry that controls addictive behaviour and motivational learning mediates this effect.	Pain chronification
Watanabe et al. [21]	2016	Western Pacific region	Japan	In the general Japanese population, the age-adjusted cross-sectional link between smoking and obesity was examined.	The prevalence of obesity did not significantly differ among the three groups of women who were divided according to their smoking habits. However, the prevalence of obesity among present and former smokers appeared to rise with pack years and daily cigarette consumption but not with time spent smoking in both genders. In addition, among males who smoked the same number of packs per year, the risk was markedly higher for short-term heavy smokers than for long-term light smokers.	The number of cigarettes smoked daily by men, including current and former smokers, may play a significant role in obesity. There was no link between smoking and obesity in women. It is advised that smoking is not helpful in preventing obesity among Japanese women.	Obesity
Chen et al. [22]	2003	Western Pacific region	China	To research the connection between smoking and liver cancer among Chinese adults who passed away from the disease.	There was a 36% increased chance of dying from liver cancer in adult men (35 years and older). This shows the absolute odds of dying from liver cancer before age 70 in the total male population to be roughly 4% in smokers and 3% in nonsmokers. (in the absence of other causes). Since most smokers also get liver cancer, the comparable risks for smokers would be roughly 33% and for nonsmokers would be 25%.	Both men and women who smoke have an increased risk of dying from liver cancer.	Liver cancer
Gajalakshmi et al. [23]	2003	Southeast Asian region	India	To investigate the mortality of men in urban and rural India by age	The death rates from medical causes for ever-smokers in the urban study region were twice as high as those for never-smokers. The dangers were high for both bidi smoking and cigarette smoking, which is the most common urban habit. A third of the higher mortality among smokers was caused by respiratory diseases, primarily tuberculosis (4/5 (4/0-5); smoking-attributed portion 61%)), a third by vascular diseases (1/8 (1/7-1/9); smoking-attributed fraction 24%), and 11% by cancer.	Half of all male deaths from tuberculosis in India are caused by smoking, which also accounts for a quarter of all middle-aged male deaths. Smoking increases the risk of developing clinical tuberculosis. (plus smaller fractions of the deaths at other ages)	Tuberculosis
Inoue-Choi et al. [24]	2017	Region of the Americas	USA	To investigate the relationship between chronic, light smoking and overall and cause-specific mortality	The mean (SD) age was 71 (5.3) years, and there were 168 140 men (57.9%). (range, 59-82 years). The majority of those who at baseline smoked between 1 and 10 CPD reported smoking much more CPD earlier in their lifetimes. However, in each age group that they smoked, 159 (9.1%) and 1493 (22.5%) of these people reported habitually smoking between 1 and 10 CPD. Consistent smokers with fewer than 1 CPD and 1 to 10 CPD showed an increased risk of all-cause death compared to never smokers. For all-cause mortality, associations were seen in both men and women and were seen across a number of smoking-related causes of death, with lung cancer showing a particularly substantial link.	Smokers with a lifetime average of 1 to 10 CPD have higher mortality risks than nonsmokers and would benefit from quitting. These findings further demonstrate that there is no degree of cigarette smoke exposure that is risk-free.	All cause and cause specific mortality
Luu et al. [8]	2022	Western Pacific region	Republic of Korea	To investigate smoking's history and its connection to cancer incidence and mortality among Korean adult males.	There were 41146 cancer fatalities and 137788 cancer cases for the 2448548 men (aged 20 or older). Six trajectory groups were identified: never smokers, ex-smokers, new smokers, decreasing light smokers, steady moderate smokers, and steady heavy smokers. Cancer risk was higher in all smoking groups. In comparison to the steady non-smokers, the steady heavy smokers had higher cancer incidence and mortality rates (hazard ratios, HR=1.53; 95% CI: 1.49-1.58 and HR=2.64; 95% CI: 2.50-2.79, respectively). The cancer-specific research revealed that smokers had higher larynx and lung cancer incidence and mortality rates than non-smokers.	Men who smoke have a higher risk of developing most malignancies, even at moderate dosages. Quitting or reducing smoking, especially at a young age, can cut cancer incidence and mortality.	Cancer
Jee et al. [9]	2020	Western Pacific region	Republic of Korea	To investigate the relationship between the amount of smoking among Korean young adult men and the risk of bladder cancer.	The follow-up period was 14.2 (median 14.3) years and 2,280,143 person-years (PY) were examined. The mean (standard deviation) age of the 161,069 participants was 34.0 (3.9) years. 263 new instances of bladder cancer were reported throughout this time (11.5/100,000 PY). The chance of getting bladder cancer was higher in all other groups of the six trajectory groups (low steady, lowering, rise and fall, high steady, rise and abrupt fall, and very high steady) than in the low steady group. The extremely high steady group had an HR of 2.83, making it the greatest risk category. In the rise and sharp decrease group, the risk of bladder cancer was 2.61 (95% CI 1.50-4.54) as well.	With the exception of the low stable group, there were few differences in bladder cancer risk according to trajectories. So, the top goal for smokers who want to reduce their risk of bladder cancer is to stop smoking.	Bladder cancer
Ott et al. [25]	2004	European region	United Kingdom	To investigate how smoking affects older people without dementia's overall cognitive function.	The average decline in the MMSE score of non-smokers was 0.03 points per year. Former smokers' adjusted decline was 0.03 points higher and present smokers' adjusted decline was 0.13 points higher than never smokers' (p 0.001). In three of the four participating studies, smoking was linked to higher rates of decline in both men and women, those with and without a family history of dementia. A considerably higher rate of decline was associated with more cigarette pack-year exposure.	Smoking may accelerate cognitive decline in the non-demented elderly.	Accelerated cognitive decline in the non-demented elderly
Torrungruang et al. [26]	2005	Southeast Asian region	Thailand	In a cross-sectional study of older Thai individuals, ascertain the impact of smoking on the severity of periodontitis.	14.4% of smokers were still smoking, 36.9% had previously smoked, and 48.7% were non-smokers. In comparison to former smokers and non-smokers, current smokers exhibited a higher proportion of plaque-containing sites, a deeper mean probing depth, and a higher mean clinical attachment level. Current smokers had probabilities of having moderate or severe periodontitis at a rate respectively, 1.7 and 4.8 times higher than those of non-smokers. Severe periodontitis was 1.8 times more common in ex-smokers than in non-smokers. Putting an end to smoking lowers your risk of developing periodontitis. When light smokers (15 packyears) gave off smoking for ten years, their chances of developing severe periodontitis returned to those of non-smokers. When they had been smoke-free for 20 years, the odds of having severe periodontitis were the same for moderate and heavy smokers (15 packs/year).	Smoking and periodontitis are strongly correlated in Thai adults.	Periodontal diseases

## Review

Smoking is linked to a higher risk of poor functional outcomes three months after an acute ischemic stroke, according to a clinical trial conducted by Matsuo et al. on 10,852 patients admitted to multicenter hospitals between July 2007 and December 2017; the study population were patients with acute stroke who had been independent before onset [2]. Patients with thrombolysed AIS comprise the research population in a randomised clinical trial conducted by Sun et al. on 4540 patients in a multicenter setting at the time of patient enrollment. The study's major finding was that young smokers with AIS had higher CVS chances than nonsmokers with AIS [12]. When compared to patients with only one diseased vessel, patients with two or three diseased coronary arteries have a two- to three-fold higher risk of suffering a major adverse cardiac event (MACE), according to a randomised controlled trial conducted on a multicenter sample of 2065 patients by Reinstadler et al. [13]. Cigarette smoking and the effectiveness of oral antiplatelet medications are both modest and unlikely to have clinical effects, according to a clinical trial conducted by Patti et al. in a multicenter with 205 patients. The study population was smokers receiving DES after ST-segment elevation MI [14].

Alotaibi et al. conducted a multicenter, randomised controlled experiment with 24 participants, demonstrating that in the early postprandial interval (0-4 h) after eating, exercise-induced reduction in postprandial triacylglycerol was larger in nonsmokers than in smokers [15]. Chen et al.'s multicentre, randomised controlled trial involved 58 patients, suggesting that smoking cessation did not improve high-density lipoprotein-induced cellular cholesterol efflux in this study's sample of coronary artery disease smokers (n = 28) and healthy smokers (n = 30) [3]. According to a randomised clinical trial conducted on 319 hospitalised patients by Aung et al., those who received the evidence-based intervention package were about three times more likely to be successful in quitting smoking than those who received standard care [4]. A randomised clinical trial conducted by Schwartz et al. on 8153 patients who attended a panel consultation suggests that hypertension decreases after smoking is stopped; here, the sample population was veterans [16].

The key finding of a randomised clinical trial conducted by Song et al. on 84 hospitalised patients was that apoB and triglyceride levels were higher in NCAD smokers than in nonsmokers. The study's participants were adults aged 40-80 who had smoked at least 10 cigarettes per day for at least 10 years [17]. According to a randomised clinical experiment conducted by Rajaee et al. on 59 hospitalised patients, quitting smoking reduces the severity of vascular disorders in those who have peripheral artery and aneurysmal disease [18]. A correlational study using cross-sectional data was conducted by Perski et al.; here, 223,537 people participated in the open-access online survey. The study's findings show that former daily smokers reported higher levels of bodily pain than non-daily smokers at all ages, indicating that smoking for a prolonged period of time may have an impact on pain perception [19]. According to a longitudinal brain imaging-based observational study conducted in a hospital setting by Petre et al., smoking increases the risk of developing chronic back pain; this effect is mediated by the corticostriatal circuitry that is involved in addictive behaviour and motivational learning. The study population in this instance is made up of patients with chronic back pain, subacute back pain (which includes back pain for 4-12 weeks and back pain without relief for at least a year), and healthy controls [20].

According to a population-based cross-sectional observational study conducted by Watanabe et al. on 23,106 persons (20-79 years of age), smoking is associated with obesity in men who are smokers. Smoking had no impact on female obesity in this study because the participants were general Japanese population smokers who were classified based on their smoking behaviours [21]. According to Chen et al.'s case-control study of 36,000 liver cancer patients and 17,000 cirrhosis patients, smoking increases both men's and women's risk of dying from liver cancer. The study population included 27000 urban and 16000 rural men who had passed away from causes other than suicide, homicide, or accidents, as well as 20,000 urban and 15000 rural male controls [22].

The results of a case-control study conducted by Gajalakshmi et al. on 43,000 adult male dead patients and 35,000 adult male controls show that smoking, which increases the incidence of clinical tuberculosis, is the cause of half the male tuberculosis deaths in India and of a quarter of all male deaths in middle age. The study population here consisted of 36,000 adults who had died from liver cancer (cases) and 17,000 who had died from cirrhosis (controls) [23]. According to a prospective cohort study on 290,000 people conducted by Inoue-Choi et al. people who have smoked one to ten cigarettes over the course of their lives have higher mortality risks than those who have never smoked and might benefit from quitting. These findings further demonstrate that there is no safe threshold for tobacco smoke exposure. In this study, 290 and 215 participants from the AARP Health and Diet Study, aged 59 to 82 years, were included. The study was conducted in 2004 and 2005 [24]. 

According to a population-based cohort study by Luu et al., smoking raises the risk of the majority of malignancies in men, even at modest doses. Smoking cessation or reduction, particularly when done at a young age, can reduce the incidence and mortality of cancer. Men who completed four health tests in 2002-2003, 2004-2005, 2006-2007, and 2008-2009 were included in their analyses, along with all covered individuals under the national health insurance plan [8]. According to a population-based cohort study conducted by Jee et al. on 430,951 people, the risk of bladder cancer did not change significantly according to the trajectory, with the exception of the low stable group. In order to reduce the risk of bladder cancer in smokers, stopping smoking should be the top priority. In this study, smoking history data were collected from the study population using a self-administered questionnaire. The history of cigarette smoking was coded from 1 to 5. The numbers 1 and 2 represent non-smokers, respectively, whereas the numbers 3 through 5 represent daily cigarette consumption of more than 20 cigarettes [9].

A prospective cohort study conducted by Ott et al. on 17,610 participants in a multicenter setting suggests that smoking may hasten cognitive deterioration in non-demented seniors, with seniors 65 and older making up the study's target demographic [25]. There is a strong correlation between smoking and periodontitis in Thai adults, according to a cross-sectional study conducted by Torrungruang et al. on 2,276 participants. The study population here consisted of senior employees and retired personnel of the Electrical Generating Authority of Thailand (EGAT) [26].

Based on these results and the numerous important discoveries from these 20 studies, we concluded that smoking is associated with an increase in cardiovascular diseases and a decrease in life quality. Hospitals and dialysis centres are where the 20 investigations were conducted. As a result, these findings are not applicable to the non-smoking community.

## Conclusions

The relationship between exposure to tobacco smoke and cardiovascular risk is nonlinear, with a steep rise at low exposure levels (including secondhand smoke exposure or infrequent cigarette smoking) and a shallower dose-response relationship as daily cigarette smoking increases. Smoking cigarettes causes a chronic inflammatory state that aids in the development of atherogenic disease processes and raises levels of inflammatory biomarkers, which are known to be excellent indicators of cardiovascular events. Nearly every organ in the body suffers damage from smoking, which also increases the risk of illness and lowers smokers' overall health. Our study clearly shows a greater prevalence of smoking, which is detrimental to patients' general health and cardiovascular systems. More research on the connections between smoking and general health and cardiovascular conditions like stroke, myocardial infarction, cardiac heart disease, peripheral artery disease, etc. suggests that these conditions reduce smokers' quality of life, increase their risk of passing away and visiting the hospital, and make it more difficult for them to recover.

## References

[1] Lugg ST, Scott A, Parekh D, Naidu B, Thickett DR (2022). Cigarette smoke exposure and alveolar macrophages: mechanisms for lung disease. Thorax.

[2] Matsuo R, Ago T, Kiyuna F (2020). Smoking status and functional outcomes after acute ischemic stroke. Stroke.

[3] Chen HY, Li SC, Chen LF, Wang W, Wang Y, Yan XW (2019). The effects of cigarette smoking and smoking cessation on high-density lipoprotein functions: implications for coronary artery disease. Ann Clin Biochem.

[4] Aung MN, Yuasa M, Moolphate S (2019). Effectiveness of a new multi-component smoking cessation service package for patients with hypertension and diabetes in northern Thailand: a randomized controlled trial (ESCAPE study). Subst Abuse Treat Prev Policy.

[5] Kondo T, Nakano Y, Adachi S, Murohara T (2019). Effects of tobacco smoking on cardiovascular disease. Circ J.

[6] Song Q, Chen P, Liu XM (2021). The role of cigarette smoke-induced pulmonary vascular endothelial cell apoptosis in COPD. Respir Res.

[7] Onor IO, Stirling DL, Williams SR (2017). Clinical effects of cigarette smoking: epidemiologic impact and review of pharmacotherapy options. Int J Environ Res Public Health.

[8] Luu MN, Han M, Bui TT, Tran PT, Lim MK, Oh JK (2022). Smoking trajectory and cancer risk: A population-based cohort study. Tob Induc Dis.

[9] Jee Y, Jung KJ, Back JH, Lee SM, Lee SH (2020). Trajectory of smoking and early bladder cancer risk among Korean young adult men. Cancer Causes Control.

[10] Konstantinou E, Fotopoulou F, Drosos A (2018). Tobacco-specific nitrosamines: A literature review. Food Chem Toxicol.

[11] Page MJ, McKenzie JE, Bossuyt PM (2021). The PRISMA 2020 statement: an updated guideline for reporting systematic reviews. BMJ.

[12] Sun L, Song L, Yang J (2021). Smoking influences outcome in patients who had thrombolysed ischaemic stroke: the ENCHANTED study. Stroke Vasc Neurol.

[13] Reinstadler SJ, Eitel C, Fuernau G (2017). Association of smoking with myocardial injury and clinical outcome in patients undergoing mechanical reperfusion for ST-elevation myocardial infarction. Eur Heart J Cardiovasc Imaging.

[14] Patti G, Polacco M, Taurino E, Gaudio C, Greco C (2016). Effects of cigarette smoking on platelet reactivity during P2Y12 inhibition in patients with myocardial infarction undergoing drug-eluting stent implantation: results from the prospective cigarette smoking on platelet reactivity (COPTER) study. J Thromb Thrombolysis.

[15] Alotaibi TF, Thackray AE, Roberts MJ (2021). Acute running and coronary heart disease risk markers in male cigarette smokers and nonsmokers: a randomized crossover trial. Med Sci Sports Exerc.

[16] Schwartz MD, Jensen A, Wang B (2015). Panel management to improve smoking and hypertension outcomes by VA primary care teams: a cluster-randomized controlled trial. J Gen Intern Med.

[17] Song W, Wang W, Dou LY, Wang Y, Xu Y, Chen LF, Yan XW (2015). The implication of cigarette smoking and cessation on macrophage cholesterol efflux in coronary artery disease patients. J Lipid Res.

[18] Rajaee S, Holder T, Indes JE (2019). A pilot study of a standardized smoking cessation intervention for patients with vascular disease. Ann Vasc Surg.

[19] Perski O, Garnett C, Shahab L, Brown J, West R (2020). Associations between smoking status and bodily pain in a cross-sectional survey of UK respondents. Addict Behav.

[20] Petre B, Torbey S, Griffith JW (2015). Smoking increases risk of pain chronification through shared corticostriatal circuitry. Hum Brain Mapp.

[21] Watanabe T, Tsujino I, Konno S (2016). Association between smoking status and obesity in a nationwide survey of Japanese adults. PLoS One.

[22] Chen ZM, Liu BQ, Boreham J, Wu YP, Chen JS, Peto R (2003). Smoking and liver cancer in China: case-control comparison of 36,000 liver cancer deaths vs. 17,000 cirrhosis deaths. Int J Cancer.

[23] Gajalakshmi V, Peto R, Kanaka TS, Jha P (2003). Smoking and mortality from tuberculosis and other diseases in India: retrospective study of 43000 adult male deaths and 35000 controls. Lancet.

[24] Inoue-Choi M, Liao LM, Reyes-Guzman C, Hartge P, Caporaso N, Freedman ND (2017). Association of long-term, low-intensity smoking with all-cause and cause-specific mortality in the National Institutes of Health-AARP Diet and Health Study. JAMA Intern Med.

[25] Ott A, Andersen K, Dewey ME (2004). Effect of smoking on global cognitive function in nondemented elderly. Neurology.

[26] Torrungruang K, Nisapakultorn K, Sutdhibhisal S (2005). The effect of cigarette smoking on the severity of periodontal disease among older Thai adults. J Periodontol.

